# Letter from Springfield

**Published:** 1883-10

**Authors:** John H. Rauch

**Affiliations:** Office of the Secretary, Springfield, Ill.


### Domestic Correspondence.

### ARTICLE XI.

OFFICE OF THE SECRETARY,
SPRINGFIELD, Ill., Aug. 13, 1883.

*Dear Sir*:—I beg to call your attention to the following, without any comment other than the remark that nowhere in my report is there any claim that the Sanitary Council has done anything more than *supervise the inspections* formerly maintained by the National Board of Health. Extract from report of proceedings of the Louisiana State Board of Health, at its regular meeting in New Orleans, August 9th, 1883:

"Mr. Booth, after eulogizing the press in general, said it sometimes assisted, whether knowingly or not, to circulate false and injurious statements. One of them was a report made by Dr. John H. Rauch, agent of the Sanitary Council of the Mississippi Valley, and published in a recent issue of the Memphis *Avalanche*.

"Mr. Booth characterized the statements and tenor of the publication to be false and libelous upon this Board, and he thought the Board should reply to it by some official act. However false such a publication, it will mislead honest people, and for that reason it should receive notice.

"Mr. Booth then offered the following:

"WHEREAS, It appears that Dr. J. H. Rauch, of Springfield, Ill., signing himself as Sec. S. C. M. V., has made a report to President Hadden, of Memphis, wherein it is stated that the late National Board of Health and the present Sanitary Council of the Mississippi Valley have so successfully conducted the Louisiana-Mississippi quarantine during 1883, that it is no longer necessary for a Sanitary Council inspector to remain at said quarantine, and that consequently said inspector had been withdrawn, with much other alleged information touching the conduct, statistics, management and general work done and action had at said quarantine, intended doubtless to misrepresent the situation in so far as to lead to the belief that this Board was not, and the Sanitary Council was, attending to the health affairs of the State and valley as they stand affected by their relation to the Mississippi River and its commerce with tropical countries. Therefore, as such belief would be based upon erroneous grounds, to the detriment, as far as entertained, of the standing of this Board before the people, be it

"*Resolved*, That this Board protests against the statements of said Secretary Rauch as a tissue of curiously interwoven statistics, in themselves true, but perverted by a malignant intelligence to the uses of falsification and perversion, incredible in persistence and monumental in audacity.

"*Resolved*, That a copy of this paper be sent to the President of the Sanitary Council of the Mississippi Valley, that he may lay it before his association at their next meeting, with a view to the removal of said Dr. Rauch from an office which he puts to so poor or so bad a use.

"The resolution was adopted.

"The Board then adjourned."

Full text of report characterized by Dr. Joseph Jones, President of the Board, and Mr. Booth, a member, as "false and libelous" in "statements and tenor:"

"Dr. Rauch, the executive officer of the Sanitary Council of the Mississippi Valley, has completed his report of the operations during the month of July, of the inspection service, formerly maintained by the National Board of Health, but now supervised by the council. From this it is learned that at the Mississippi river quarantine station, below New Orleans, there have arrived, during the quarantine season, seventy-three vessels from foreign ports. Of these, forty-seven were inspected up to June 30 under the supervision of the National Board of Health, and during July the remaining twenty-six were inspected under the supervision of the sanitary council."

"Of these eleven were from ports infected by yellow fever at the date of departure, and three of them—namely, the Berna, July 3, the Merchant, July 16, and the Buteshire, July 17, arrived with cases of yellow fever on board. Among the remaining vessels one was found to have had yellow fever on board in Havana last season, and in seven other cases it was probable that they had been infected at some previous time."

"The sanitary condition of the vessel, crew, cargo, and passengers in twelve cases was good, and in the remaining vessels, with the exception of the Berna, Merchant, and Buteshire, which were infected, the report of the inspector was qualified. In all cases the vessels were subjected to a thorough general cleansing, purification of bilge, hold, etc., and disinfection with carbolic acid and copperas, and the cargoes were fumigated with sulphurous acid gas."

"Coffee ships from Rio de Janeiro were either not allowed to proceed up to New Orleans at all, or only after removal of cargo and thorough fumigation of the same."

"The arrival of the Merchant on the 16th of July, and of the Buteshire on the following day, both from Vera Cruz, with yellow fever cases, finally led the governor of Louisiana, on the 20th of July, to recommend to the Louisiana State Board of Health that no infected vessel be permitted to enter the Mississippi river, and that all infected vessels then at the quarantine station be removed out of the river at once, assigning the reason that their presence at that point had practically rendered the station an infected port, in dangerous proximity to New Orleans, and threatened a stupendous calamity to the Mississippi. At its meeting on July 23, the Board discussed this communication, and, on the 24th, Gov. McEnery issued a proclamation enforcing his recommendations, and declaring non-intercourse between Louisiana and Vera Cruz, Rio de Janeiro, Havana, and other infected ports."

"The infected vessels have been removed to Ship Island, and, for the first time in a number of years, the lower Mississippi is freed from the menace arising from the admission of yellow fever ships to the river."

"Immediately upon receipt of information of this action, the request, previously referred to Governor McEnery to permit the Sanitary Council inspector to remain at the quarantine station, was withdrawn by telegram, and the inspector was relieved from duty as soon as the infected vessels and yellow fever patients were removed."

"It is remarked in this connection that while the National Board of Health and the Sanitary Council have been advocating for the past four years the exclusion of infected vessels from the Mississippi during the dangerous season, and the use of Ship Island as a refuge station for such vessels, the necessity for absolute non-intercourse has not been recognized. The plan proposed by these two bodies contemplated the maintenance of an inspection station at or near Port Eads. All vessels entering the river would here be subjected to a rigid examination. Those found to be infected would be compelled to go to the Ship Island refuge station; healthy vessels from infected ports would be subjected to such treatment as would render it safe to allow them access to New Orleans; while all others, if found in a good sanitary condition, would be passed without detention. In this way it was believed the public health could be properly protected without inflicting such serious injury to the commercial interests of New Orleans as a non-intercourse quarantine necessarily entails.

The executive committee of the Council, however, does not feel warranted in criticising any action which promises to secure the safety of the valley from an invasion of yellow fever.

During the month forty-five steamboats and other river craft, with an aggregate capacity of 44,219 tons, and carrying 2,532 officers, crews and passengers, were inspected at New Orleans, and furnished with the certificates of the Sanitary Council. On the Illinois Central and Louisville and Nashville railroads, at the same point, 144 freight trains bound northwise, and their crews, comprising 1,194 persons, were also inspected.

At the inspection stations at Fort Adams, Miss., and President's Island, Tenn., all north-bound vessels are now inspected, as well as those plying in the local trade and those clearing from New Orleans. The total inspections for the month at these stations comprise 123 steamboats, tows, barges, etc., carrying 5,352

persons. The sanitary condition of these vessels is reported as satisfactory, and an entire freedom from illness of a suspicious nature is noted.

An aggregate of 338 ocean vessels, river craft and freight trains, together with 9,342 officers, crews and passengers, has been inspected under the supervision of the Council during the month.

I send you this as a fair illustration of the methods and animus of the Louisiana State Board of Health. Very truly yours,

JOHN H. RAUCH.

To the Editor of the MEDICAL JOURNAL AND EXAMINER,
Chicago.

---

MACALINA.

A new remedy has been discovered, and, by the experiments already made, it seems to equal quinine in the treatment of malarial fevers. This new remedy is macalina, an alkaloid extracted by Dr. Doudé from the bark of a tree known in Yucatan as yaba. According to Dr. Rosado, the sulphate of macalina cures intermittent fevers in the same dose as quinine, and he prefers it to the latter for its surety and absence of symptoms (it only produces transient pains in the abdomen), and for being almost tasteless, and easily taken by children. It is to be hoped that the experiments will be continned, so as to ascertain if macalina can be considered equal to quinine.—*Il Morgagni.*

ANTISEPTIC CATGUT.

According to Kocher a truly antiseptic cat-gut is prepared by placing it for twenty-four hours in pure juniper oil, and then kept in 95 per cent. alcohol. This catgut is not absorbed before the sixth or seventh day. If the cat-gut is desired flexible, and not rapidly dried when exposed to the air, it is to be placed in glycerine for a day before being conserved in the alcohol.—*Lo Sperimentale.*

